# The Role of Fundoscopy Before Lumbar Puncture in Children with Suspected Meningitis

**DOI:** 10.1007/s00431-025-06603-w

**Published:** 2025-11-14

**Authors:** Nir Friedman, Or Kaplan, Gidon Test, Jordanna H. Koppel, Gal Altberg, Neta Cohen, Nitai Levy, Segev Gil-ad, Naama Kuchinski Cohen, Nili Yanai Milshtein, Hagit Poran Feldman, Haitam Mreisat, Yulia Gimelraikh, Gal Kimhi, Liat Gelernter Yaniv, Ron Jacob

**Affiliations:** 1 Pediatric Emergency Department, Meir Medical Center, Kfar Saba, Israel; 2 Pediatric Emergency Department, Saban Pediatric Medical Center, Soroka University Medical Center, Beer Sheva, Israel; 3 Pediatric Emergency Department, Sheba Medical Center, Tel Hashomer, Ramat Gan, Israel; 4 Emergency Department, Schneider Children’s Medical Center, Petah Tikva, Israel; 5 Pediatric Emergency Department, Dana Dwek Children’s Hospital, Tel Aviv Sourasky Medical Center, Tel Aviv, Israel; 6 Pediatric Emergency Department, Rambam Health Care Campus, Haifa, Israel; 7 Pediatric Emergency Department, Hadassah-Hebrew University Medical Center, Jerusalem, Israel; 8 Pediatric Emergency Unit, Hillel Yaffe Medical Center, Hadera, Israel; 9 Pediatric Emergency Department, Samson Assuta University Hospital, Ashdod, Israel; 10 Pediatric Emergency Department, Laniado Sanz Medical Center, Natania, Israel; 11 Pediatric Emergency Unit, The Tzafon Medical Center, Poriya, Israel; 12 Pediatric Emergency Department, Shaare Zedek Medical Center, Jerusalem, Israel; 13 Pediatric Emergency Department, Mayanei Hayeshua Medical Center, Bnei Brak, Israel; 14 Pediatric Emergency Unit, Bnai-Zion Medical Center, Haifa, Israel; 15 Pediatric Emergency Department, Ha’Emek Medical Center, Yitzhak Rabin Boulevard 21, 1834111 Afula, Israel; 16 Gray Faculty of Medicine and Health Science, Tel Aviv University, Tel Aviv, Israel; 17 Adelson School of Medicine, Ariel University, Ramat HaSharon, Israel; 18 Azrieli Faculty of Medicine, Bar Ilan University, Ramat Gan, Israel; 19 The Ruth and Bruce Rappaport Faculty of Medicine, Technion-Institute of Technology, Haifa, Israel; 20 Faculty of Health Science, Ben Gurion University of the Negev, Beer Sheva, Israel

**Keywords:** Meningitis, Pediatric emergency department, Fundoscopy, Lumbar puncture

## Abstract

**Purpose:**

To assess the diagnostic yield and clinical impact of fundoscopy before lumbar puncture (LP) in children with suspected meningitis.

**Methods:**

A multicenter retrospective cohort study across 15 pediatric emergency departments, including children aged 18 months–18 years with suspected meningitis between July 2018 and June 2023. The primary outcome was papilledema prevalence; secondary outcomes were subsequent abnormal neuroimaging, LP deferral, and time to antibiotics.

**Results:**

Among 1,742 children (median age 4.6 years), bacterial meningitis was confirmed in 56 (3.2%). Fundoscopy was performed by ophthalmology residents in 959 (55.1%). Papilledema was identified in 27 (2.8%); all underwent CT, which revealed no contraindications to LP, and all proceeded to LP. Three children with normal fundoscopy who underwent LP were later found to have CT abnormalities (brain abscess and tumor); none of which led to adverse outcomes. Antibiotics were administered before LP less often when fundoscopy was performed (42.6% vs 58.8%, p < 0.001), although median time to antibiotics was similar (341 vs 269 min, p = 0.52). Children who had a head CT without fundoscopy received antibiotics earlier (239 vs 341 min; p = 0.039), and more frequently before LP (64.3% vs 42.6%, p < 0.001).

*Conclusions*: Routine fundoscopy before LP rarely detected papilledema, did not identify contraindications to LP, and was associated with lower rates of early antibiotic treatment. These findings suggest that fundoscopy adds little to safe LP decision-making and may inadvertently delay care in time-critical meningitis. A selective, clinically guided pre-LP approach, rather than routine fundoscopy, could streamline evaluation and improve practice.

**Table:** 

**What is known:** *• Fundoscopy is often performed before lumbar puncture (LP) in children with suspected meningitis to detect papilledema and guide decision about neuroimaging.* *• Evidence for its diagnostic yield and clinical impact in pediatric emergency settings is limited.*	
**What is new:** *• In a multicenter cohort of 1,742 children, fundoscopy (performed by ophthalmology residents) detected papilledema in only 2.8% of cases and revealed no contraindications to LP.* *• Fundoscopy was associated with lower rates of early antibiotic administration.*

## Introduction

Lumbar puncture (LP) is essential for the diagnosis of meningitis but carries a risk of cerebral herniation in patients with raised intracranial pressure (ICP) [1–3]. To reduce this risk, clinical guidelines recommend evaluating for signs of raised ICP prior to LP. Papilledema, detectable via fundoscopy, is traditionally considered a key indicator of raised ICP, and may lead to deferral of LP and use of neuroimaging [4–9].

In pediatric emergency care, fundoscopy is frequently performed during the initial evaluation of children with suspected meningitis. In our study, several institutional protocols recommend routine fundoscopy for all such cases, typically performed by ophthalmology residents in the pediatric emergency department (PED). However, the utility of this practice remains unclear. Papilledema may not appear until several days after ICP begins to rise [10], and its absence can offer false reassurance. Conversely, its detection can prompt additional imaging, potentially delaying diagnosis and treatment, despite limited supporting evidence.

Guidelines discourage routine head Computed Tomography (CT) before LP in children unless clinical signs of raised ICP are present [4–8] due to risks of radiation exposure, procedural delay, and limited ability to exclude herniation risk [1, 11]. Nevertheless, fundoscopy findings are often used to guide imaging decisions, introducing variability into practice and potential delays in antibiotic administration—a critical factor in bacterial meningitis outcomes [12].

Despite its widespread use, the diagnostic yield and clinical impact of fundoscopy in this setting have not been systematically evaluated. We aimed to assess the prevalence of papilledema and examine whether fundoscopy influences imaging use or delays antibiotic treatment in children with suspected meningitis.

## Methods

### Study design and setting

This multicenter, retrospective cohort study was conducted across 15 public hospitals in Israel between July 1, 2018, and June 30, 2023. These hospitals account for approximately 90% of all PED visits nationwide. The study was coordinated by the Israel Pediatric Emergency Research Network (ISPERN), and was approved by the Institutional Review Board of Ha’Emek Medical Centre (EMC – 0095–23). Each of the 14 other participating hospitals also obtained local ethics approval prior to data collection.

### Participants and eligibility criteria

Children aged 18 months to 18 years presenting to the PED with suspected meningitis were eligible. Suspected meningitis was determined by the treating clinician’s documentation in the medical record (clinical notes), rather than by administrative coding, to ensure inclusion reflected clinical judgement. The lower age limit was chosen to exclude infants with open fontanelles. Exclusion criteria were ventriculoperitoneal shunts, known central nervous system (CNS) masses, documented papilledema prior to presentation, LP or fundoscopy performed for indications other than suspected CNS infection, and transfer from another emergency department or urgent care center.

### Case identification

At each site, two lists were generated from electronic medical records: (1) children who underwent LP and (2) children who underwent fundoscopy followed by head imaging without LP. To capture potential LP deferrals prompted by papilledema detection, the second group included all ophthalmology consultations requested in the PED to assess for papilledema during the index visit; only encounters with clinical notes consistent with suspected meningitis were included. Records were screened by site investigators using uniform criteria. Children were eligible regardless of whether an LP was ultimately performed or deferred during the index visit. Included patients were centrally reviewed by two independent investigators (RJ and NF). Discrepancies were resolved by consensus.

### Data collection

Trained reviewers extracted data using a standardized case report form, including demographics (age, sex), visit details (arrival and discharge times, disposition), presenting symptoms (fever, headache, vomiting, photophobia, seizures) and duration, physical examination findings (meningeal signs, shock, petechial rash, signs of Cushing’s triad, altered mental status, lateralizing signs), and diagnostic procedures (fundoscopy findings and timing, neuroimaging findings and timing, LP results including culture and PCR). Treatment and outcomes, including timing of antibiotic administration and mortality, were also recorded.

### Definitions

A 48-h cutoff from hospital arrival to LP was used to capture investigations initiated during the initial diagnostic workup. Fundoscopy-positive papilledema cases leading to neuroimaging and LP deferral were included if this occurred during the initial evaluation. Raised ICP on clinical examination was defined as the presence of Cushing’s triad (hypertension, bradycardia, irregular respiration) and/or abnormal pupillary findings (anisocoria, fixed or non-reactive pupils), representing severe manifestations of increased ICP. Other symptoms potentially associated with raised ICP, such as headache, vomiting, or focal neurological signs, were recorded separately but not included in this definition. Abnormal neuroimaging was defined as findings suggestive of raised ICP (midline shift, herniation, effacement of CSF spaces, or cerebral oedema), mass lesion, or other diagnoses contraindicating LP. LP deferral was defined as clinician decision not to perform LP during the index visit. Time to antibiotics was defined as the interval from PED arrival to first documented intravenous antibiotic dose, recorded in minutes.

### Outcomes

The primary outcome was the prevalence of papilledema among children undergoing fundoscopy. Secondary outcomes included prevalence of abnormal neuroimaging following papilledema detection, LP deferral, and timing of antibiotic administration. We also compared children who underwent CT before LP without prior fundoscopy with those who underwent fundoscopy before LP.

### Statistical analysis

Continuous variables are reported as medians (IQRs) and categorical variables as frequencies (percentages). Adjusted odds ratios (aORs) with 95% CIs were calculated using multivariable logistic regression to identify factors associated with undergoing CT before LP without prior fundoscopy versus fundoscopy before LP, and with study outcomes. Statistical significance was set at p < 0.05. Analyses were performed in R version 4.3.1. Cases with missing key data for a given variable were excluded from the corresponding analysis.

## Results

A total of 1,742 children met inclusion criteria (median age 4.6 years [IQR 2.9–8.0]; 1,072 [61.5%] male) (Table 1). Most presented with fever (1,329 [76.3%]), vomiting (1,151 [66.1%]), or headache (1,002 [57.5%]); meningeal signs were documented in 979 (56.2%). Bacterial meningitis was confirmed in 56 children (3.2%), and one patient died.

**Table 1:** Baseline characteristics of children evaluated for suspected meningitis in the PED (*N* = 1,742)

Variable	Value
Age, years, median (IQR)	4.6 (2.9–8)
Sex, No. (%)	
Male	1072 (61.5)
Female	670 (38.5)
Admission, No. (%)	1559 (89.5)
Admission to PICU, No. (%)	160 (9.2)
Presenting symptoms, No. (%)	
Fever	1329 (76.3)
Vomiting	1152 (66.1)
Headache	1002 (57.5)
Seizures	273 (15.7)
Photo/phonophobia	220 (12.6)
Symptom duration, days, median (IQR)	2 (1–3)
Clinical Examination Findings, No. (%)	
Meningeal signs	976 (56.2)
Decreased LOC	283 (16.3)
Focal neurological signs	236 (13.6)
Poor perfusion	97 (5.6)
Purpura	79 (4.5)
Signs of increased ICP	19 (1.1)
Meningitis, No. (%)	
Bacterial	56 (3.2)
Viral	441 (25.3)

*PED* pediatric emergency department, *IQR* interquartile range, *No.* number, *min* minutes, *LP* lumbar puncture, *PICU* pediatric intensive care unit, *LOC* level of consciousness, *ICP* intra cranial pressure

Fundoscopy was performed in 959 children (55.1%). Papilledema was detected in 27 (2.8%), all of whom subsequently underwent CT. None had neuroimaging findings that contraindicated LP. Among children with bacterial meningitis, 1 had papilledema on presentation, which resolved on follow-up within one week. Seven children with papilledema had notable CT findings (e.g., venous sinus thrombosis, mastoiditis, sinusitis), none of which precluded LP by imaging criteria. All children with papilledema subsequently underwent LP following neuroimaging.

Three children with normal initial fundoscopy were later found to have CT-detected lesions (two brain abscesses, one astrocytoma). However, none of these children developed clinical signs of herniation, and all were eventually discharged home.

CT without prior fundoscopy was performed in 496 children (28.4%). A further 287 children (16.5%) were evaluated clinically without fundoscopy or CT.

When comparing children who underwent fundoscopy with those who did not, median time to antibiotic administration was 341 min (IQR 191–552) vs 269 min (IQR 129–528; p = 0.52), and antibiotics were given before LP in 42.6% vs 58.8% of cases (p < 0.001).

Children who underwent fundoscopy were generally less severely ill than those who did not, with higher rates of fever (80.8% vs 70.8%), vomiting (71.0% vs 60.2%), and headache (65.7% vs 47.5%), but lower rates of seizures (9.8% vs 22.9%), decreased level of consciousness (10.5% vs 23.2%), focal neurological signs (9.1% vs 19.0%), and signs of raised ICP (0.4% vs 1.9%) (all p < 0.001, except signs of raised ICP p = 0.003).

Compared with children who had fundoscopy prior to LP, those who had CT prior to LP without fundoscopy had higher rates of seizures (33.3% vs 7.9%), decreased level of consciousness (23.9% vs 8.5%), focal neurological signs (27.7% vs 7.6%), and clinical signs of raised ICP (4.4% vs 0.2%) (all p < 0.001). Antibiotics were administered before LP in 64.3% of the CT group versus 41.1% of the fundoscopy group (p < 0.001), with a shorter median time to antibiotic administration (239 min [IQR 97–500] vs 341 min [197–544], p = 0.039) (Table 2).

In multivariable logistic regression, older age (aOR 1.06, 95% CI 1.03–1.09), seizures (aOR 2.58, 95% CI 1.87–3.57), focal neurological signs (aOR 2.79, 95% CI 2.02–3.85), and signs of raised ICP (aOR 8.49, 95% CI 2.23–56.48) were independently associated with undergoing CT before LP without prior fundoscopy. Fever (aOR 1.87, 95% CI 1.47–2.38), headache (aOR 1.56, 95% CI 1.22–2.00), and meningeal signs (aOR 1.29, 95% CI 1.04–1.60) were independently associated with undergoing fundoscopy before LP (Table 3).

**Table 2:** Comparison of Pre-LP assessment pathways: fundoscopy vs. CT imaging

	CT (*n* = 339)	Fundoscopy (*n* = 802)	*p*-Value
Age, years, median (IQR)	4.2 (2.5–8.8)	4.6 (3–7.6)	0.361
Sex, N (%)			0.148
Male	198 (58.4)	505 (63.0%)	
Female	141 (41.6%)	297 (37.0%)	
Antibiotic treatment before LP, N (%)	178 (64.3%)	265 (41.1%)	< 0.001
Time to Antibiotics, min, median (IQR)	239 (97–500)	341 (197–544)	0.039
Presenting symptoms, N(%)			
Fever	256 (75.5)	668 (83.3)	0.002
Vomiting	188 (55.5)	578 (72.1)	< 0.001
Headache	155 (45.7)	534 (66.6)	< 0.001
Seizures	113 (33.3)	63 (7.9)	< 0.001
Photo/phonophobia	46 (13.6)	108 (13.5)	0.963
Symptom duration, days, median (IQR)	2 (1–3)	2 (1–3)	0.105
Clinical Examination Findings, N(%)			
Meningeal signs	143 (42.6)	535 (66.8)	< 0.001
Decreased LOC	81 (23.9)	68 (8.5)	< 0.001
Focal neurological signs	94 (27.7)	61 (7.6)	< 0.001
Poor perfusion	25 (7.4)	32 (4.0)	0.0162
Purpura	17 (5.0)	39 (4.9)	0.917
Signs of increased ICP	15 (4.4)	2 (0.2)	< 0.001

*IQR* interquartile range, *N* number, *min* minutes, *LP* lumbar puncture, *PICU* pediatric intensive care unit, *LOC* level of consciousness, *ICP* intra cranial pressure

**Table 3:** Multivariable logistic regression: clinical predictors of fundoscopy and ct use prior to lumbar puncture

	Fundoscopy (aOR [95% CI])	CT (aOR [95% CI])
Age	0.99 [0.96–1.01]	1.06 [1.03–1.09]
Male sex	0.93 [0.76–1.15]	0.87 [0.69–1.09]
Presenting symptoms		
Fever	1.87 [1.47–2.38]	0.77 [0.60–1.01]
Vomiting	1.21 [0.97–1.51]	0.96 [0.75–1.22]
Headache	1.56 [1.22–2.00]	0.97 [0.73–1.30]
Seizures	0.63 [0.45–0.86]	2.58 [1.87–3.57]
Photo/phonophobia	0.82 [0.60–1.12]	1.50 [1.06–2.09]
Symptom duration	0.97 [0.94–1.01]	1.07 [1.03–1.11]
Clinical Examination Findings		
Meningeal signs	1.29 [1.04–1.60]	0.70 [0.55–0.89]
Decreased LOC	0.51 [0.38–0.69]	1.78 [1.30–2.43]
Focal neurological signs	0.47 [0.34–0.64]	2.79 [2.02–3.85]
Poor perfusion	0.73 [0.47–1.13]	1.46 [0.91–2.31]
Purpura	0.85 [0.52–1.37]	0.72 [0.39–1.28]
Signs of increased ICP	0.65 [0.18–1.89]	8.49 [2.23–56.48]

*IQR* interquartile range, *N* number, *min* minutes, *LP* lumbar puncture, *PICU* pediatric intensive care unit, *LOC* level of consciousness, *ICP* intra cranial pressure

## Discussion

This large multicenter cohort study is, to our knowledge, the largest to date—and the first—to specifically evaluate the role of fundoscopy in the pre–LP assessment of children with suspected meningitis. Although not mandated as a universal screening tool, fundoscopy is recommended in some guidelines as part of the assessment for papilledema, which is considered an indication for neuroimaging before LP [4–6, 8, 9, 13, 14]. In our cohort, fundoscopy was routinely performed by ophthalmology residents in the PED, representing a higher level of examiner expertise than in most pediatric emergency settings. Despite this, papilledema was detected in only 2.8% of cases, and no subsequent neuroimaging demonstrated findings that contraindicated LP. Thus, even when performed by relatively skilled examiners, the diagnostic yield and impact of fundoscopy were limited. In some cases, neuroimaging performed after papilledema detection identified other intracranial pathologies such as venous sinus thrombosis, mastoiditis, or sinusitis; however, none of these constituted formal contraindications to LP, and all affected children ultimately underwent the procedure safely. In children with meningitis, papilledema was transient and later reclassified as normal. By contrast, clinical signs such as seizures, decreased level of consciousness, and focal neurological deficits—well-recognized guideline-based triggers for imaging—were the primary reasons for CT use. Notably, children who underwent fundoscopy examination had fewer markers of acute neurological severity than those without fundoscopy, suggesting that illness severity may have influenced the choice of pre-LP assessment.

Our findings are consistent with prior studies questioning the reliability and clinical utility of fundoscopy for detecting papilledema in pediatric emergency settings, even when performed by trained clinicians [15, 16]. In pediatric patients, the sensitivity of fundoscopy is influenced by examiner expertise [17], patient cooperation, and the time course of intracranial pressure elevation. Papilledema may develop days after the onset of raised ICP [10], and its absence should not be considered reassuring in suspected meningitis. Conversely, its detection can prompt neuroimaging that delays diagnosis and treatment, despite CT’s limitations in ruling out raised ICP and the possibility of meningitis-related ICP elevation without radiological signs [18]. No cases of cerebral herniation occurred in our study, supporting earlier evidence that careful clinical assessment can safely guide LP decisions without routine pre-procedure imaging [19].

Our findings have implications for how pre-LP assessment is organized in pediatric emergency settings. While the median time to antibiotics did not differ significantly overall (likely reflecting wide variability), pathway-specific differences were evident. Children who underwent fundoscopy were significantly less likely to receive antibiotics before LP. In contrast, those evaluated with initial CT without fundoscopy more often received early antibiotics and had shorter median times to treatment. These patterns may reflect the added procedural step and subsequent imaging in the fundoscopy pathway, although causation cannot be confirmed, and could also be influenced by differences in underlying illness severity. Given the established link between delayed antibiotics and worse outcomes in bacterial meningitis [12, 20, 21], even small delays merit consideration. CT was used more selectively, and its shorter time to antibiotics likely reflects urgent clinical decision-making. These observations underscore that fundoscopy should not delay stabilization, empiric antibiotic therapy, or indicated neuroimaging, although it may retain value when there is specific concern for vision-threatening papilledema or markedly raised intracranial pressure.

Our findings support a streamlined approach to pre-LP assessment in pediatric emergency care. Selective imaging based on guideline-supported indications—such as seizures, decreased level of consciousness, focal neurological deficits, or other signs of raised ICP—should remain the primary trigger for neuroimaging, with fundoscopy considered in situations where the results are likely to guide further diagnostic evaluation rather than a routine. Although neuroimaging after papilledema detection occasionally revealed other pathologies (e.g., venous sinus thrombosis, mastoiditis, sinusitis), these findings did not represent formal contraindications to LP, and all affected children underwent the procedure safely. Importantly, our study was designed to evaluate the diagnostic yield of fundoscopy in relation to LP decision-making, rather than its role in detecting alternative intracranial diagnoses. This approach may help minimize potential delays and ensure timely treatment in children with suspected meningitis.

### Limitations

This study has several limitations. Its retrospective design carries the risk of documentation and classification biases. Fundoscopy was performed by ophthalmology residents, which may limit generalizability to settings where the examination is done by less experienced clinicians. However, this likely biased our study toward greater sensitivity, suggesting that the low diagnostic yield observed may be an overestimate compared with typical PED practice. Glasgow Coma Scale scores were not available, and decreased level of consciousness was used as a proxy, which may have limited precision in assessing mental status. Timing discrepancies between clinical deterioration, imaging, and LP decisions could not be fully accounted for. Because fundoscopy and CT were ordered at clinician discretion, selection by illness severity likely influenced group comparisons despite adjustment, introducing residual confounding. Furthermore, lumbar puncture opening pressure was not routinely measured in the participating centers and could therefore not be analyzed. Finally, practice patterns in our institutions may differ from those in other health systems, which could limit generalizability.

## Conclusion

In this large multicenter cohort of children with suspected meningitis, fundoscopy before LP identified few cases of papilledema and no subsequent neuroimaging findings that contraindicated the procedure. Although still used in many centers, its limited clinical contribution and potential to delay antibiotics raise questions about the value of routine fundoscopy in this context. Our findings support prioritizing timely clinical evaluation and selective imaging guided by established indications, rather than performing routine fundoscopy before lumbar puncture.

## Data Availability

The datasets generated and/or analysed during the current study are not publicly available due to institutional and ethical restrictions but are available from the corresponding author on reasonable request.

## Abbreviations

LP: Lumbar puncture
CT: Computed tomography
ICP: Intra cranial pressure
PED: Pediatric emergency department
CNS: Central nervous system
CSF: Cerebrospinal fluid
IQR: Interquartile range
aOR: Adjusted odds ratio
CI: Confidence interval

## Code availability

N/A.
